# Editorial commentary on “urological age and mortality risk: an investigation of urological age acceleration”

**DOI:** 10.1097/JS9.0000000000002350

**Published:** 2025-03-18

**Authors:** Cyrille Guillot-Tantay, Mikolaj Przydacz

**Affiliations:** a Department of Urology, Hopital Foch, Suresnes, France; b Department of Urology, Jagiellonian University, Krakow, Poland

*Dear Editor*,

The article titled “Urological Age and Mortality Risk: An Investigation of Urological Age Acceleration” introduces a novel approach to assessing urological health through the concept of “urological age” (UA) and its acceleration (UAA), utilizing laboratory parameters and age-related symptoms^[^1^]^. This study, based on the NHANES dataset, offers an intriguing correlation between urological age acceleration and mortality, suggesting that UAA can be a useful screening tool for identifying individuals at heightened risk of multi-system ageing and mortality. While this research offers a promising avenue for precision medicine in ageing interventions, several points merit further scrutiny to enhance the validity and applicability of the findings.

One of the key strengths of this study is its attempt to quantify urological health through a systematic, evidence-based approach that could provide insights into the broader mechanisms of ageing. The use of the Klemera Doubal method to determine UAA is an innovative adaptation that merges laboratory findings with age-related urological symptoms, offering a potential framework for clinical practice^[^2^]^. The Klemera and Doubal method is a mathematical approach to estimating biological age based on the relationship between chronological age and multiple biomarkers. Unlike traditional multiple linear regression models, which often lead to imprecise biological age estimates, this method treats chronological age as a standard biomarker, significantly improving accuracy.

The high correlation between UA and chronological age (r = 0.85 for men and 0.84 for women) underscores the robustness of the model in estimating the urological component of ageing.

Furthermore, the study’s finding that UAA is associated with higher mortality risks—specifically the 1% increase for men and 4% for women per year of UAA—adds valuable evidence to the growing body of literature that links urological health with broader health outcomes. These results could help clinicians identify high-risk patients and offer early intervention strategies to mitigate the effects of ageing on the urological system, as well as multi-system dysfunction.

While the study contributes valuable insights, several limitations should be addressed. First, the method of categorizing UAA into discrete grades (0, 1, and 2) might oversimplify the complex nature of ageing. Urological function and its decline may not follow such a linear progression, and a more nuanced, continuous scale could provide a more precise understanding of how urological age relates to both functional decline and mortality risk. Future studies should explore alternative ways of categorizing UAA that account for the variability and individual differences observed in ageing processes.

Additionally, the study’s reliance on cross-sectional data from NHANES and the West China Natural Population Cohort Study raises concerns regarding causality. While the correlation between UAA and mortality risk is clear, the directionality of this relationship remains uncertain. Longitudinal studies that track changes in UAA over time, alongside other biomarkers of ageing, would provide more robust evidence of causality and the potential benefits of early interventions.

Moreover, the role of confounding factors such as comorbidities, socioeconomic status, and access to healthcare should be considered. While the study controls for some of these variables, it is unclear how these factors may have influenced the results, particularly given the diversity of the NHANES cohort. Understanding the interplay between these variables and urological age acceleration would be crucial in refining the clinical utility of UAA as a predictive tool for mortality.

The clinical implications of this study are substantial. If UAA can be reliably used as a predictor of mortality, it could revolutionize the way clinicians approach preventative care for ageing populations. A better understanding of urological ageing could lead to more personalized treatments and interventions aimed at delaying the onset of urological dysfunction and associated comorbidities. Furthermore, the association between UAA and healthy lifestyle choices is an important finding that could help shape public health guidelines, encouraging preventative strategies that include diet and exercise to mitigate the acceleration of urological ageing.

However, for UAA to be integrated into clinical practice, more research is needed to validate its predictive power across different populations and healthcare systems. Comparative studies that examine UAA in diverse ethnic and socio-economic groups will be crucial for establishing its generalizability. Additionally, the potential for UAA to be incorporated into routine screening protocols would require a cost-benefit analysis to ensure its practicality and cost-effectiveness in primary care settings.

This research lays an important foundation for future studies and could significantly contribute to the development of interventions aimed at improving the quality of life and longevity for ageing populations.
